# Trabecular Level Analysis of Bone Cement Augmentation: A Comparative Experimental and Finite Element Study

**DOI:** 10.1007/s10439-012-0587-3

**Published:** 2012-05-31

**Authors:** Y. Zhao, K. A. Robson Brown, Z. M. Jin, R. K. Wilcox

**Affiliations:** 1 Institute of Medical and Biological Engineering, School of Mechanical Engineering, University of Leeds, Leeds, LS2 9JT UK; 2 Imaging Laboratory, Department of Archaeology and Anthropology, University of Bristol, Bristol, UK; 3 School of Mechanical Engineering, Xi’an Jiaotong University, Xi’an, 710054 China

**Keywords:** Bone cement interface, Vertebroplasty, Validation, Multiscale

## Abstract

The representation of cement–augmented bone in finite element (FE) models of vertebrae following vertebroplasty remains a challenge, and the methods of the model validation are limited. The aim of this study was to create specimen-specific FE models of cement–augmented synthetic bone at the microscopic level, and to develop a new methodology to validate these models. An open cell polyurethane foam was used reduce drying effects and because of its similar structure to osteoporotic trabecular bone. Cylindrical specimens of the foam were augmented with PMMA cement. Each specimen was loaded to three levels of compression inside a micro-computed tomography (μCT) scanner and imaged both before compression and in each of the loaded states. Micro-FE models were generated from the unloaded μCT images and displacements applied to match measurements taken from the images. A morphological comparison between the FE-predicted trabecular deformations and the corresponding experimental measurements was developed to validate the accuracy of the FE model. The predicted deformation was found to be accurate (less than 12% error) in the elastic region. This method can now be used to evaluate real bone and different types of bone cements for different clinical situations.

## Introduction

Percutaneous vertebroplasty is a minimally invasive treatment for osteoporotic vertebral fractures. During the treatment, polymethylmethacrylate (PMMA) bone cement is injected into the fracture site to augment the fracture and relieve pain. The success of the treatment is still under debate, with clinical trials reporting conflicting results in terms of pain relief.^2^,^10^,^11^ There is also evidence to suggest that the cement–bone composite region affects the load distribution through the vertebrae, and further fractures may occur in the adjacent vertebrae after the procedure.^3^,^14^

The behavior of the cement–bone interface is likely to be an important factor in determining the performance of the procedure in the longer term.^5^,^17^ An increasing number of studies are using finite element (FE) methods to evaluate mechanical aspects of vertebroplasty. The majority of these use continuum level models where the size of the finite elements is larger than that of the individual bone trabeculae. Within these models, the cement–bone composite has often been approximated by a region with the properties of pure cement.^13^,^18^ However, this approach was shown to provide poor agreement in terms of stiffness when specimen-specific augmented vertebra models were directly compared with corresponding experimental tests.^17^ Other researchers have derived properties from separate experimental data and used a reduced elastic modulus for the cement–bone region.^1^ In a recent study,^20^ specimen-specific models of cement–augmented synthetic bone specimens were developed. Here, the properties of the bone and cement composite regions were represented as inhomogeneous materials with properties derived from micro-computed tomography (μCT) scans. This study found good agreement between the model predictions and corresponding experimental data when the modulus of the cement composite region was considerably lower than that which would be expected from a “rule of mixtures” calculation. This could be because of the lack of bonding between the two materials, which has been shown previously to contribute to the resultant mechanical properties,^5^ or because of localized buckling or failure of the trabecular struts around the boundary of the cement region.

In order to investigate these possible mechanisms, higher resolution models are necessary. A number of authors have previously developed micro finite element (μFE) models of trabecular structures based on μCT images (e.g., studies by van Rietbergen *et al*.^15^ and Wolfram *et al*.^19^), but there remains a challenge in validating the results of such studies. Methods have been developed to image bone specimens under load and determine the strain fields by image registration with the specimens in the unloaded case, but as yet this method has only been validated against FE predictions, rather than the other way around.^4^

While some studies have represented the cement–bone interface in greater detail,^8^,^16^ a region of trabecular bone around the cement has yet to be represented at the level of the individual trabeculae.

The aim of this study was to develop a methodology that could be used for generating and validating specimen-specific FE models of the bone–cement composite at the trabecular level, to allow the mechanical behavior at the cement boundary to be examined in more detail. The effects of different image segmentation methods and interaction properties of the cement–bone interface were examined. A new method of validation was developed, in which the specimens were imaged under load and the deformed trabecular morphology directly compared with corresponding FE models.

## Materials and Methods

### Experimental Tests

**Figure 1:**
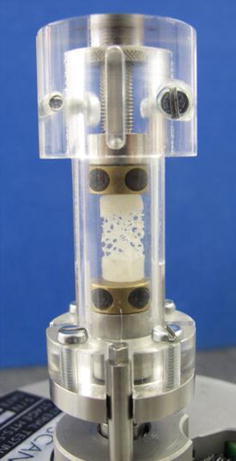
A cement–augmented specimen inside the compression stage used during μCT imaging

Since this study was primarily concerned with the development of a new methodology, a synthetic bone substitute was used to reduce handling and drying effects during preparation, and more easily allow the sectioning of small specimens. An open cell rigid polyurethane foam (pcf 7.5, Sawbone, Sweden), with porosity over 95%, cell size between 1.5 and 2.5 mm and wall thickness between 0.15 and 0.3 mm, was chosen since its structure is similar to that of human osteoporotic trabecular bone.^6^ A series of cylindrical specimens (diameter ~6 mm, height ~12 mm) were machined from the foam block, which was immersed in water and pre-frozen to minimize damage during cutting. In order to provide flat parallel surfaces for testing, and to form an interface between the synthetic bone and the cement, both ends of each specimen were filled completely with Cranioplastic PMMA cement (Cranioplastic, Codman Type 1-Slow set, DePuy CMW, UK) to a depth of approximately 2–4 mm. The cement was hand mixed according to the manufacturer’s instructions. All specimens were left for over 48 h before testing. Following a visual inspection, four specimens with no apparent damage to the trabecular structure were selected for testing. Each specimen was loaded at 2 μm/s in three incremental compression steps, with strains of approximately ~3, ~5, and ~15%, using a compression stage (Material Testing Stage MTS-50N) inside a micro-computed tomography scanner (SkyScan 1172, Belgium) (Fig. 1). The specimens were imaged at 25 μm isotropic voxel size before compression and after each loading step. Each scan had a duration of approximately 5 min. During the compression, the top and bottom of the loading stage moved toward each other such that the center point of the specimen remained in approximately the same location once the top of the specimen touched the top of the stage. For each loading increment, the μCT images were exported to an image processing software package (ScanIP, Simpleware, UK) for analysis.

### Computational Modeling

#### Definition of the Boundary Conditions

In order to replicate the experimental test conditions in the FE model, the displacements of both ends of the specimens were calculated from the μCT data. From the pre-loaded images, the coordinates of a set of points from both the top and bottom surface of each specimen were recorded according to their positions relative to the tube edge. The coordinates of the points in images from each of the subsequent loading increments were measured using the same method and the relative displacement calculated. More than five points were compared at each end of each specimen and the average difference between the coordinates was determined. The calculated displacements were subsequently applied to the FE models.

#### Image Segmentation

All the μCT scans were converted to grayscale images and down-sampled from 25 μm to 50 μm using image processing software (ScanIP version 3, Simpleware Ltd, UK). This resolution was able to capture the detailed morphology and maintain the connectivity of the synthetic bone trabeculae while reducing the size of the final FE models. Threshold values were chosen from the pre-loaded images by visual observation to segment the cement and synthetic bone regions. For one of the specimens (Specimen 1), a manual method was used to further refine the segmentation. Here, local modifications were implemented by visually comparing the regions with the underlying images on each individual slice to provide a more representative segmentation of the two regions.

#### Finite Element Model Generation

For each specimen, a μFE model with element size 50 × 50 × 50 μm was generated from the down-sampled pre-loaded μCT images using commercial software (ScanFE version 3, Simpleware Ltd, UK). The cement and synthetic bone materials were meshed as separate parts and all surfaces of the model were smoothed using tetrahedral elements. The total number of elements for each model was approximately 3.2–3.6 million. For Specimen 1, two models were generated, one from the standard “automated” segmentation method and a second from the manual segmentation method.

#### Material Properties

The material properties of both the synthetic bone and cement were considered to be homogenous and linearly elastic, with elastic moduli of 280 and 2280 MPa^9^, respectively. The Poisson’s ratio was assumed to be 0.3 for both materials.^17^

#### Processing

All of the models were processed using ABAQUS 6.81 (ABAQUS Inc, Providence, RI, USA). A high specification computer (Intel^®^ Xeon^®^ CPU X5472 @ 3.00 GHz 2.99 GHz, 32 GB memory) was used for the analysis.

### Validation

In previous studies of bone strain mapping, image registration methods have been used to compare the strain between images of strained specimens and FE models.^4^ This method was not directly applicable to the current study due to the inclusion of tetrahedral elements, whose nodes were distributed irregularly over the surface of the FE model. Instead, a new method was developed to allow direct comparison between the model-predicted deformed morphology and that seen in the μCT scans of the loaded specimen.

**Figure 2:**
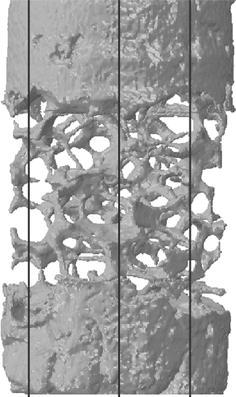
The vertical slices taken from different sagittal positions

Prior to generating the models, markers were manually added to the μCT images at specific locations using the “paint” function within the image processing software. These were then included in the model generation process to enable a registration area in the model outputs to be compared with the same area in the μCT images. Vertical slices were then taken at three locations distributed through the FE model (Fig. 2) and scaled to the same resolution as the μCT images. Direct comparisons between the corresponding slices from the μCT images and μFE model were then made. The difference between the model-predicted deformed morphology and that seen in the μCT scans was quantitatively assessed by comparing the pixels in the registered area between the two images. For each pixel, if the FE image value represented either the cement or trabecular bone region and the μCT pixel represented background, the pixel was labeled as a “false positive,” conversely if the FE pixel represented background but the μCT image pixel was within the solid structure, this was labeled “false negative.” The comparisons were implemented using an in-house code (Matlab 7.2, MathsWorks Inc, MA, USA).

The method was first applied to the unloaded specimen images and corresponding unloaded model. This step was undertaken to calibrate the error measurements and evaluate the differences due to the model generation and smoothing processes. The comparison was then undertaken on the images and models from each loading increment. The total error was calculated as the sum of the false positive and false negative pixels as a fraction of the total number of pixels in the trabecular and cement regions.

In addition, the local stress distributions were examined in the FE models, and the deformations at specific locations around the interface between the cement and the synthetic bone were compared to the μCT images at each loading increment.

### Sensitivity Studies

For Specimen 1, a comparison of the predicted stiffness and stress was undertaken between the manual and automated segmentation models. The difference in the predicted deformations between the two FE models was evaluated at three locations through the models using the method described in “Validation” section.

The effect of the different interaction properties used at the interface between the cement and the synthetic trabeculae was also studied using the manually segmented model of Specimen 1. Contact between cement and synthetic bone was modeled using a small sliding, node to surface algorithm and three different interaction properties were examined: tied, frictionless, and with a coefficient of friction of 0.3. The stiffness and the maximum stress in the models were compared between the three cases. In addition, the local effects of using different contact algorithms on the predicted deformed morphology was quantified between the two extreme cases (tied and frictionless) using the method described in “Validation” section.

## Results

### Validation

**Figure 3:**
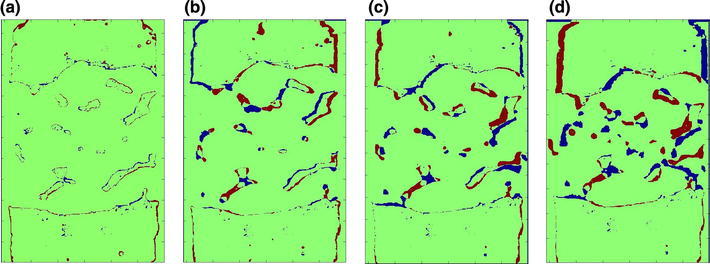
Comparison between the FE predicted deformation and the μCT image at: (a) the initial position, (b) Load Increment 1, (c) Load Increment 2, and (d) Load Increment 3. The green region indicates that FE images matches μCT image, while the blue regions represents “false negative” and red region represents “false positive” pixels

The difference between the predicted deformations seen on the FE slices and the μCT images for the manual segmentation model is shown in Fig. 3. A high level of agreement was found at low strains, but this decreased at the higher levels of applied displacement.

**Figure 4:**
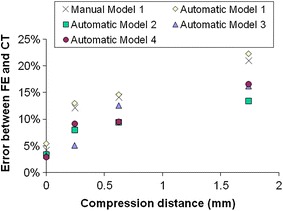
Error between FE predicted deformations and actual deformation measured using μCT for the manually and automatically segmented models

Similar results were found for the automated segmentation models, as shown in Fig. 4. The average percentage errors in the initial position and at each loading increment over the whole area of the trabeculae and cement region are given in Table 1.

**Table 1:** Difference between the FE-predicted deformation and the corresponding μCT image over the area of synthetic bone and cement (average of five models)

Difference in deformed morphology	False positive Mean (SD) (%)	False negative Mean (SD) (%)	Total error Mean (SD) (%)
Initial	2.25 (1.18)	1.57 (0.29)	3.82 (1.04)
Step 1	5.73 (1.83)	3.74 (1.49)	9.47 (3.20)
Step 2	6.55 (1.43)	5.48 (1.15)	12.03 (2.50)
Step 3	9.92 (1.82)	7.95 (1.95)	17.87 (3.66)

**Figure 5:**
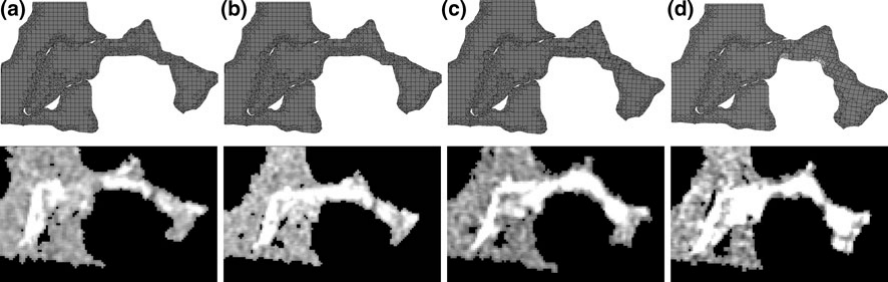
Local deformation for a single synthetic bone “trabecula” at the cement–trabeculae interface as predicted in the FE models and as observed in the μCT images at (a) The initial position, (b) Load Increment 1, (c) Load Increment 2, and (d) Load Increment 3

The predicted deformations near the interface between the cement and synthetic bone for the FE models are shown in Fig. 5, along with corresponding μCT images.

### Sensitivity Studies

For Specimen 1, the difference in stiffness, maximum stress and deformed morphology between the manual segmentation model and the automated segmentation model are shown in Table 2. The difference in stiffness and morphology was found to be small (less than 1%), but the maximum von Mises stress was considerably lower in the manual segmentation model (approximately 20% less).

**Table 2:** Difference in the predicted stiffness, maximum von Mises stress, and deformed morphology (averaged over three locations) between the models of Specimen 1 generated using the manual and automated segmentation methods

	Stiffness	Maximum von Mises stress	Morphology
Difference (%)	+0.77	−19.4	+0.49

The values shown are the maximum difference (manual–automated) found across all compression steps calculated as a proportion of the result from the manual segmentation model

**Figure 6:**
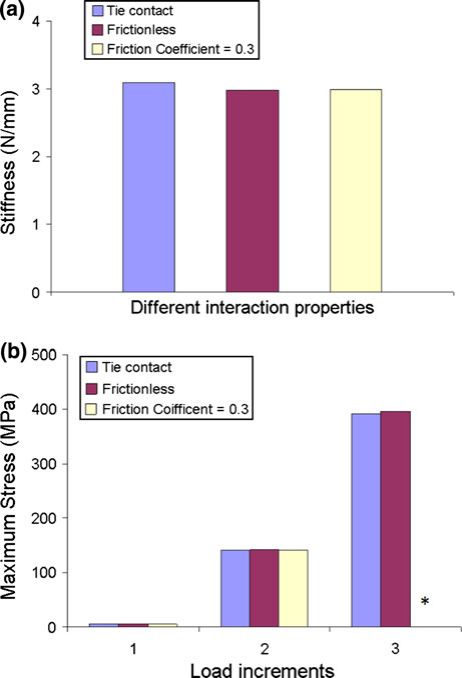
Comparison between the three contact algorithms in terms of (a) the stiffness and (b) the maximum von Mises stress at each load increment. *The case where the friction coefficient = 0.3 was stopped before Load Increment 3 due to computational restrictions

For the manual segmentation model, the effects of using different interaction properties (tied, frictionless, and with a coefficient of friction 0.3) at the interface between the cement and the synthetic trabeculae in terms of the stiffness and the maximum von Mises stress are shown in Fig. 6. The maximum difference in the stiffness using different interaction properties was found to be 3.47%, and the difference in maximum stress was less than 1%.

The difference in the predicted deformed morphology between the tied and frictionless models in the registration areas at different load increments was found to be very small; with the maximum difference after the final compression step less than 0.7% (Table 3).

**Table 3:** Difference in the predicted deformed morphology between the tied contact FE model and the frictionless contact FE model

	Initial	Step 1	Step 2	Step 3
Difference (%)	0.046	0.048	0.28	0.68

**Figure 7:**
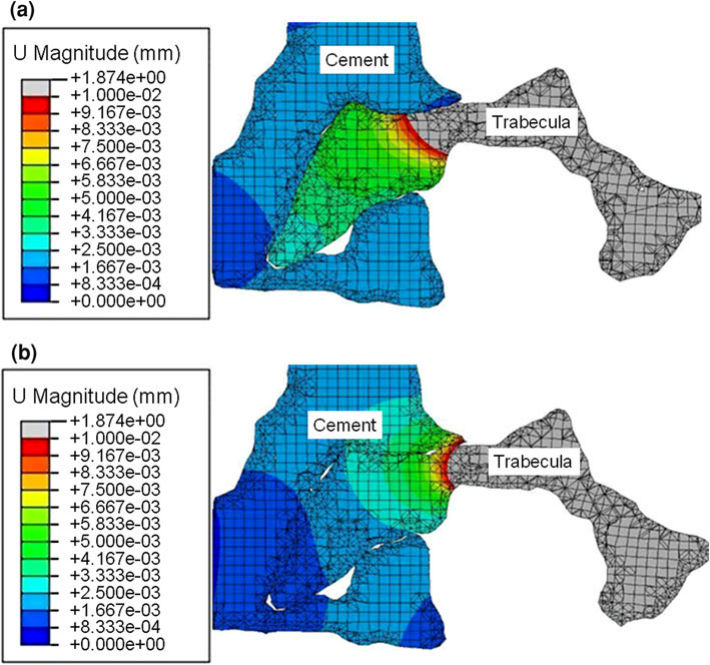
Comparison of the deformation after Load Increment 3 for (a) the frictionless model and (b) the tied contact model. This shows the relative displacement is larger near the interface between the trabecula and cement in the frictionless model than tied contact model

A comparison of the localized deformation between the tied and frictionless contact models in the cement–trabecular interface region is shown in Fig. 7. Overall, there was little difference in the deformation, although there was some relative displacement between the cement and trabeculae in the frictionless case.

## Discussion

In this study, specimen-specific μFE models of cement–augmented synthetic bone were generated to represent the structure at the trabecular level. Previous continuum level models of similar structures have been relatively successful in capturing the overall specimen behavior when a reduced modulus is applied to the cement–composite region.^20^ However, the reasons for this mechanical behavior are not fully understood and it is therefore difficult to infer from these results how other cement materials might behave. Since continuum models represent the experimental or clinical situation at a larger scale, the distribution of stress and strain are averaged over the element region and the interactions at the interfaces between the different materials are not represented. Trabecular level models can represent the cement–bone interdigitation and local motion at a far greater level of detail, so they should theoretically provide more information on the interface mechanics and how this affects the larger-scale performance. However, this method presents a number of challenges and few studies have, as yet, been undertaken. Trabecular level FE models necessitate large numbers of elements, and the computational resource required is further increased by the inclusion of contact interactions at the cement–bone interface. For example, in this study, the models were processed using a high performance computer with four parallel CPUs and 32 GB RAM, however, the running wall clock time for the least time-consuming model, which included the simplest surface interaction properties, was about 20 h. For the most time consuming model, which included a coefficient friction at the surfaces, the running time increased to over 700 h. This limited the size of structure that could be represented, although future increases in computational performance will reduce this shortcoming.

In the current study, the specimen size was also limited by the dimensions of the loading stage in the μCT. This led to difficulties in clamping the ends of the specimens, and some slipping and twisting occurred during the compression which could not be fully replicated in the FE models, accounting for some of the error seen in the validation. In order to generate a boundary region between the cement and synthetic bone in these specimens, rather than injecting cement into the center of the specimen, the ends were embedded into cement. This approach was used because of the size of the specimen and also to generate flat, parallel surfaces necessary for fixation into the loading stage. The limitation of this approach was that the cement boundary was perpendicular to the loading axis, so was only loaded in compression. In a vertebroplasty procedure, the cement is injected into the vertebral body forming a bolus surrounded by trabecular bone, so around the boundary of the cement there are different relative loading directions. The methodology presented here could now be applied to other configurations of cement to examine the boundary behavior under alternative modes of loading.

In order to generate the FE models, it was necessary to segment the μCT images into the synthetic bone and cement regions. However, there was some overlap in the grayscale distributions of the two materials. Therefore, further local manual modifications were implemented on one of the specimens using visual comparisons with the underlying images on each slice to provide a more representative segmentation of the two regions. The manual modifications preserved better connectivity of the synthetic bone trabeculae within the composite region and visually looked more accurate. However, from the FE modeling results (Table 2), the difference between the manual segmentation model and the automated segmentation model in the terms of the predicted stiffness and morphology was found to be small. The maximum stress of the manual segmentation model was 19% lower than that of the automated segmentation model, which probably is due to the smoother interfaces between the two regions in the manual segmentation with fewer sharp corners. For multiple specimens, the manual segmentation procedure would become prohibitively time consuming due to the large number of images that would need to be processed. In this case therefore, providing that the local stress is not of primary interest, or an error of the order of 20% could be tolerated, the automated segmentation method appears sufficient. The distribution of grayscale values will be different for other material combinations and, clearly, the larger the gap between the grayscales, the more accurate the resulting segmentation is likely to be.

In this study, the effect of the friction and interactions at the interface were examined using the manual segmentation model, by varying the contact interaction properties from frictionless, to a frictional coefficient of 0.3, and to a tied contact. Some differences between the tied and frictionless contacts were observed in the local deformations (Fig. 7) at the cement boundary, however the friction and interactions at the interface were found to not be critical to the overall stiffness, maximum stress, or the deformed shape of the trabeculae even close to the boundary (Fig. 6; Table 3). This finding indicates that the interlocks of cement and bone dominate the micro-mechanics at the cement boundary. It should be emphasized that in this study, compression loading dominated at the interface and the effect of friction is likely to become more significant in tension or shearing, where the interface has been shown to be more compliant.^12^ Also, in this study, dry synthetic bone was used and there may be larger gaps at the interface in the case of real bone when the cement must displace marrow. A previous FE study found a larger effect of using different interaction properties at the cement–bone interface,^7^ which is most likely due to the different loading mode used, as well as some differences in level of model detail.

A new validation method was developed in this study by examining the difference in the deformed morphology between the FE models and the experimental images. The FE models and the μCT images were compared at the initial unloaded position as a calibration (Fig. 3a) to gauge the level of error with this method (Table 1). This initial difference was found to be relatively small (~3–5%). It was caused by the model generation procedures such as the selection of threshold values and the smoothing processes. This difference could be slightly reduced by tuning the threshold value and altering the number of smoothing iterations. Good agreement was found between FE models and μCT images at low strains (Figs. 3b, 3c), indicating that the FE models were representing the experimental deformation. However, the difference tended to increase at higher strains, especially in the middle of the FE models—the region of pure synthetic bone (Fig. 3d). This was likely due to the synthetic bone trabeculae in the physical model exceeding their elastic limit, whereas in the FE models, the synthetic bone material was assumed to be linearly elastic.

At the region of the interface between the cement and Sawbone, a localized comparison between the FE model and the μCT images at each loading step (Fig. 5) showed good agreement, demonstrating that the FE model can represent the experimental situation at this interface. To improve the accuracy of the model, especially at larger strains, a modulus reduction or element deletion method could be considered to extend the model to represent the plastic and failure behavior.

## Conclusions

This study developed a method of generating specimen-specific μFE models of cement augmented synthetic bone at the microscopic level, to represent the *in vitro* cement–bone interface under load. A method of validating the models against μCT images of the same specimen under the same loading conditions by comparison of the deformed morphology was also developed. The focus of this study was on compressive loading, with the longer term aim of improving the understanding of the mechanics of cement augmented vertebra after percutaneous vertebroplasty. The effect of interaction properties at cement–bone interface was found to be minimal in compression, where the interlocks of cement and bone dominate the micro-mechanics at the cement boundary. This method will now be applied to real bone augmented with different types of bone cements used for vertebroplasty. Although this study focussed on vertebral augmentation, the modeling and validation methods developed could be used in other loading cases, such as shear or tension, to evaluate other applications such as total joint replacement.
